# Optimising mothers’ health behaviour after hypertensive disorders of pregnancy: a qualitative study of a postnatal intervention

**DOI:** 10.1186/s12889-022-13590-2

**Published:** 2022-06-27

**Authors:** Chris Rossiter, Amanda Henry, Lynne Roberts, Mark A. Brown, Megan Gow, Clare Arnott, Justine Salisbury, Annette Ruhotas, Angela Hehir, Elizabeth Denney-Wilson

**Affiliations:** 1 Susan Wakil School of Nursing and Midwifery, Faculty of Medicine and Health, The University of Sydney, Sydney, NSW 2006 Australia; 2 Discipline of Women’s Health, Faculty of Medicine and Health, The University of New South Wales, Kensington, NSW 1466 Australia; 3 Department of Women’s and Children’s Health, St George Hospital, Kogarah, NSW 2217 Australia; 4 St George and Sutherland Clinical School, The University of New South Wales, St George Hospital, Kogarah, NSW 2217 Australia; 5 Department of Renal Medicine, St George Hospital and the University of NSW, Sydney, Australia; 6 The University of Sydney Children’s Hospital Westmead Clinical School, Westmead, NSW 2145 Australia; 7 The George Institute for Global Health, The University of New South Wales, 1 King Street, Newtown, NSW 2042 Australia; 8 Department of Cardiology, Royal Prince Alfred Hospital, Camperdown, NSW 2050 Australia; 9 NSW Ministry of Health, 1 Reserve Road, St Leonards, NSW 2065 Australia; 10 Consumer and Community Involvement Representative, Sydney, Australia; 11 Sydney Local Health District, Royal Prince Alfred Hospital, Camperdown, NSW 2050 Australia

**Keywords:** Hypertensive disorders of pregnancy, Pregnancy-induced hypertension, Heart disease risk factors, Pre-eclampsia, Health behavior, Healthy diet, Exercise, Healthy lifestyle, Clinical trial, Qualitative research

## Abstract

**Background:**

Hypertensive disorders of pregnancy have longer-term implications, increasing women’s lifetime cardiovascular disease risk. The Blood Pressure Postpartum study is a multi-centre randomised three-arm trial of interventions, ranging in intensity and including education and lifestyle coaching, to support women to maintain or adopt healthy eating and physical activity during the first postpartum year. This qualitative sub-study nested within the main trial aimed to investigate whether and how women adopted healthy behaviours after a pregnancy complicated by a hypertensive disorder.

**Methods:**

Semi-structured telephone interviews were recorded, transcribed and analysed thematically, following Braun and Clarke principles. They explored behaviour change among new mothers following their hypertensive pregnancy, and the intervention’s effect on their capacity and motivation to pursue healthy lifestyles.

**Results:**

Thirty-four women from all three trial arms participated at 10–12 months postpartum. The three main themes were 1) Awareness of cardiovascular risk: some did not acknowledge the health risks, whereas others embraced this information. 2) Sources of motivation: while the majority were motivated to make a concerted effort to adapt their health behaviour, motivation often centred on their baby and family rather than their own needs. 3) Sustaining behaviour change with a new baby: women in the more intensive intervention arm demonstrated increased recognition of the importance of reducing cardiovascular health risks, with greater motivation and guidance to change their health behaviour. There was minimal evidence of crossover amongst groups, with women largely accepting their randomised level of intervention and not seeking additional help when randomised to minimal intervention.

**Conclusions:**

Among women participating in an early post-hypertensive disorders of pregnancy randomised controlled trial aimed at improving their cardiovascular disease risk profile, the majority recognised the future health risks and appeared motivated to improve their lifestyle, particularly women in the highest-intensity intervention group. This highlights the importance of structured support to assist women embrace healthy lifestyles especially during the challenges of new parenthood.

**Trial registration:**

The Blood Pressure Postpartum study was prospectively registered as a clinical trial with the Australian New Zealand Clinical Trials Registry (anzctr.org.au) on 13 December 2018 (registration number: ACTRN12618002004246).

**Supplementary Information:**

The online version contains supplementary material available at 10.1186/s12889-022-13590-2.

## Background

Hypertensive disorders of pregnancy (HDP) complicate 5–10% of pregnancies [1], including preeclampsia (2–5%), gestational hypertension (3%) and chronic hypertension (1–2%) [2]. HDP not only affect women during pregnancy but have implications for their ongoing health, doubling the lifetime risk for cardiovascular disease (CVD) [3–7], as well as Type 2 diabetes and kidney disease [5, 8]. The lifelong risk is as high – or higher – than that of well-recognised CVD risk factors such as smoking [7]. Infants born to women with HDP are also at greater risk of cardiometabolic disorders, experiencing higher blood pressure and weight through childhood and adolescence than other children [9, 10]. Australian and international guidelines [11, 12] recommend follow-up, annual blood pressure checks, regular assessment for CVD risk factors, in addition to counselling to avoid smoking and to maintain a healthy weight with regular exercise and a healthy diet. In practice, these recommendations are rarely translated systematically into clinical care as some healthcare professionals have limited awareness of guidelines for best practice postnatal care [13–15].

Despite the established link with subsequent CVD, there is limited evidence from interventions aimed at women following HDP [16]. Moreover, Australian women have limited knowledge of how to maintain health after HDP [13, 17] and follow-up programs are inadequate in helping them address risk factors and adopt healthier lifestyles [18]. Previous studies have identified that participants were generally eager to increase their understanding of HDP, and several expressed interest in lifestyle interventions to reduce their risk of future HDP and ongoing CVD [19, 20]. Small-scale trials after preeclampsia suggest women’s health behaviours improve following structured lifestyle behaviour change interventions, although the extent and the downstream impact on women’s CVD risk measures remain unclear [21, 22].

Previous research on more general postnatal lifestyle behaviour change interventions has indicated some success in improving health outcomes. A 2013 meta-analysis reported higher average weight reduction from four randomised trials of postnatal diet plus physical activity interventions among women with overweight or obesity [23]. A 2015 meta-analysis of 46 studies identified that postpartum lifestyle interventions focusing on diet-and-exercise modifications were more effective in promoting weight loss and related outcomes in the first year than physical activity alone, especially if combined with self-monitoring [24]. A qualitative study of a 12-week weight management program for postnatal women with obesity or excessive gestational weight gain identified barriers to uptake and retention. These included limited opportunity to attend face-to-face group sessions, a poor understanding of the program plan, or limited motivation; support from the intervention (staff and peers), partners and family enhanced retention [25]. However, the recently published LIVING randomised trial of a 12-month lifestyle intervention following gestational diabetes did not halt worsening of glycaemic status in the 1601 randomised participants, [26] so lifestyle intervention impact following medical pregnancy complications such as HDP and gestational diabetes is far from assured.

The Blood Pressure Postpartum Study (BP^2^) is a multicentre three-arm randomised controlled trial (RCT) of follow-up and lifestyle behaviour change strategies during the first year after HDP [27]. Interventions range from optimised usual care (following expert recommendations for follow-up) through to extended lifestyle interventions (described below). This paper reports a qualitative sub-study nested within the main BP^2^ study, to explore women’s adoption of healthy lifestyles after pregnancies complicated by HDP.

This sub-study aims to examine the impact of the study interventions on women’s health behaviour following HDP.

## Methods

### Research design

This qualitative study used semi-structured interviews with RCT participants.

### Intervention

The BP^2^ study included women who had experienced an HDP, recruited during their admission to give birth or in the first six months postpartum at five hospitals in Sydney, Australia, collectively serving a socio-demographically diverse population with nearly 19,000 births annually. Research nurses/midwives approached eligible women, provided participant information and enrolled those who expressed immediate interest; at five months postpartum they confirmed participation with those who had consented and offered enrolment to other eligible women. Participants were randomised into three groups: 1) Optimised Usual Care supplementing standard care of six-weeks postnatal visit with information brochures from the study and general practitioner (GP) follow-up six months postpartum for best-practice follow-up as recommended by SOMANZ and international guidelines [11, 12], 2) Brief Education Intervention (information brochures plus a consultation with a dietitian and physician in specialised clinic six months postpartum) or 3) Extended Lifestyle Intervention (Brief Education Intervention plus referral to a six-month lifestyle coaching service, the Get Healthy Service, delivered every 2–3 weeks via telephone. https://www.gethealthynsw.com.au/). Women completed anthropometric assessment and surveys regarding their current lifestyle as well as physical and mental health at six months postpartum, when the two interventions (2 and 3) commenced. The primary study outcomes are systolic blood pressure, and lifestyle change as measured by weight and waist circumference change between six and 12 months postpartum (primary outcome assessment at 12 months postpartum). Further details of the trial are included elsewhere [27].

### Ethics

The study was approved by the South Eastern Sydney Local Health District human research ethics committee (2019/ETH04732). BP^2^ was prospectively registered as a clinical trial (ACTRN12618002004246).

### Recruitment

In the six-month BP^2^ survey, all participants were invited to an optional telephone interview when their infants were approximately 10 months old. The researchers recruited purposefully to ensure equivalent numbers from each intervention group. Interested participants received information about the qualitative sub-study and consent forms. The BP^2^ Project Manager obtained informed consent, and then passed limited details (given name, phone number, infant gender, hospital and intervention group) to the interviewer, who arranged a convenient time for the interview. Three infants were nearly 12 months by the interview, given delayed responses and complex scheduling. Women received a gift voucher for AUD$30 to compensate for their time.

### Interviews

Semi-structured interviews were conducted via telephone and audio-recorded with participants’ consent. The interviewer [initials] is a female social scientist, not involved in other aspects of the BP^2^ trial, with experience in qualitative research. The schedule was developed by the BP^2^ research team which included a consumer representative, informed by the Social Ecological Model [28] which recognises that behaviour is determined by the multi-layered and inter-connected effects of personal and environmental factors. Questions focused on participants’ diet and eating habits, their physical activity, other health behaviours and their contact with health professionals (including BP^2^ clinicians). The interview thus addressed barriers and facilitators to healthy eating and physical activity related to each layer: individual (knowledge, attitudes and behaviours), relationship, community, and societal factors. Questions also addressed participants’ motivations and their experiences of the BP^2^ program (see Supplementary material for interview guide). The interviewer pilot-tested the questions and adjusted the order. She kept field notes after the telephone interviews.

The interviews ranged from 18 to 47 min in length and were conducted between March 2020 and April 2021, when data saturation was achieved, as no new themes were apparent in the responses to the last few interviews. The interview period included government ‘stay at home’ orders across Sydney due to COVID-19 outbreaks.

### Data analysis

Interviews were professionally transcribed, cleaned and identifying information removed. The researcher aimed to validate the data by offering to return transcripts to the participants to check for accuracy and resonance with their experience; none accepted this offer. Transcripts were imported into NVivo qualitative data analysis software (QSR International) for data management. Data were analysed using the thematic approach outlined by Braun and Clarke [29], consisting of deep familiarisation with the data; complete coding across the dataset in relation to the research question; searching for themes across the coded data; reviewing, defining and naming the themes; and finalising the analysis. The first author performed most analysis, assisted by senior author and authors [initials], who all reviewed transcripts and were involved in discussing and developing codes, themes and sub-themes until agreement was reached to validate the findings. Themes and sub-themes are presented below, illustrated by typical excerpts from participants.

## Results

### Sample

We interviewed 34 BP^2^ participants: 12 from Group 1 and 11 each from Groups 2 and 3. This represents 25% of those who were willing to receive further information about the qualitative sub-study. Table 1 summarises their demographic characteristics compared with all BP^2^ participants at the time of recruitment.

**Table 1:** Sample characteristics compared with BP.^2^ cohort

	**Interviewees** ***N* = 34**			**All BP**^**2**^** participants** **to February 2021** ***N* = 157**		
	**n**	**%**	**Mean (SD)**	**n**	**%**	**Mean (SD)**
**Age (years)**			33 (5.3)			33 (5.2)
**Country of birth**						
Australia	26	76		96	61	
Not Australia	8	24		61	39	
**Ethnicity**						
Caucasian/European	30	88		100	64	
Asian	3	9		23	15	
African	0	0		6	4	
Polynesian	0	0		4	3	
Middle Eastern	1	3		3	2	
Aboriginal and/or Torres Strait Islander	0	0		1	< 1	
Other/mixed ethnicity	0	0		10	6	
Ethnicity not stated	0	0		10	6	
**Highest level of education**						
Secondary school	4	12		21	13	
Trade certificate/diploma	9	26		40	25	
University degree	21	62		87	56	
No data available	-	-		9	6	
**Parity**						
First baby	29	85		96	61	
Subsequent baby	5	15		61	39	
**Plurality**						
Singleton	32	94		154	98	
Twins	2	6		3	2	
**HDP**						
Chronic hypertension (CH)	2	6		30	19	
Gestational hypertension (GH)	9	26		45	28	
Preeclampsia (PE)	20	59		73	47	
Chronic hypertension with superimposed preeclampsia	3	9		9	6	
**Gestation at birth (weeks)**			37 (2.6)			37 (2.6)
≥ 37^+0^ weeks	26	76		123	78	
34^+0^—36^+6^ weeks	5	15		20	13	
< 34^+0^ weeks	3	9		14	9	

Compared to the BP^2^ cohort overall, the interviewed sample had higher proportions with only one child, Caucasian ethnicity and born in Australia. Interviewees were comparable in maternal age, gestational age and educational attainment. Compared with the wider population of women giving birth in New South Wales, Australia during 2020, women in the BP^2^ study were slightly older but similar proportions were Australian-born and had multiple births. There were fewer Aboriginal or Torres Strait Islander women. The rate of premature birth was understandably higher in the study group [30].

### Themes

In addressing the research question, we identified three themes in responses about the role of trial participation in facilitating healthy behaviour changes. Table 2 shows themes and related sub-themes.

**Table 2:** Data analysis—themes and sub-themes

Themes	Sub-themes
Awareness of cardiovascular risk: “It makes you more high risk”	New knowledge from BP^2^ – “I didn’t know that before” Stressful information – “Knowing is scary” Awareness doesn’t necessarily change behaviour – “I know what I’m meant to do”
Sources of motivation to change behaviour: “Who am I changing for?”	My future health – doing it for me Baby/family as motivation – doing it for them My needs come last – certainly not doing it for me Putting good health on hold – not doing much
Sustaining behaviour change with a new baby: “I do what is comfortable for me and with the time I've got”	Adapting to new circumstances Value of BP^2^ and the Get Healthy Service Taking what is given/need for structured follow-up

#### Theme 1: awareness of cardiovascular risks: “It makes you more high risk”

Despite the importance of HDP as a risk factor for subsequent cardiovascular disease, not all interviewees recognised the significance of maintaining healthy lifestyles post-pregnancy. Apart from monitoring their blood pressure, few interviewees recalled discussing cardiovascular health with their GP. Certainly, several mothers in Groups 2 and 3 became more aware of the risks due to study participation, sometimes as their only information source.

“It wasn't until I went for that first meeting for the study that they said that you should make sure your GP checks in on these things, since you've had preeclampsia.”

M07, Group 2, primipara

“Especially when we spoke about the increased risks of other medical issues that can come about … I’m realizing that it's so important to just go back to being healthy to reduce any second chance of preeclampsia and just all those other risks that come along with it.”

M20, Group 2, primipara

“I was not aware of the risk consequences of preeclampsia … I thought, ‘Okay, you have it, and then... you have the baby, it subsides, and that's it’… That education was really helpful ... I'm aware of the risks. I'm aware of what I can do to take control which, if I hadn't participated in the study, I wouldn't have known.”

M34, Group 3, primipara

Some mothers in Group 1 reported that study involvement increased their knowledge, although this seemed more often from the BP^2^ brochures than GP consultation:

“I did get that fact sheet that said … It’s like seven times more likely to have it again and all that … I suppose the doctors don't want to freak you out, but I think that's the first time I kind of saw the stats of like it makes you more high risk, all these things”

M01, Group 1, primipara

Some interviewees reported finding the new information about longer term cardiovascular risks worrying.

“That's [6-month visit for BP^2^] how I found that all the risk of heart disease and stroke, and I'm like ‘Oh my God’."

M25, Group 3, primipara

“Having young kids at a later stage in life, I guess is a bit scary because they're only young, and I'm 41, which means I need to be healthier to be here for them for a lot longer.”

M10, Group 2, primipara

Conversely, some mothers did not seem to recognise the significance of HDP for their ongoing health, despite having access to health professionals through the intervention. This group included mothers who continued in unhealthy behaviours despite stating that they were aware of the importance of a healthy diet and exercise from either the BP^2^ program or other health providers.

“I haven't really thought about it too much… If anything, the only thing that stuck with me was my salt intake. I probably have tried to address that… I wouldn’t be saying I’ve been trying hard, but I’ve been conscious of it… I told her I eat five chocolate biscuits a day and she said ‘well, you just need to think about that’… so there’s nothing else really, I think, that I didn’t already kind of know”

M11, Group 2, primipara

One woman did not identify as having had HDP, possibly illustrating the hidden nature of this condition to those who do not have immediate or severe complications, and consequently did not read the information brochures or take other action.

“I don't consider myself having high blood pressure. I was more than happy to partake in the research, but I don't feel like I have high blood pressure, so it hasn't really made a difference … I don't know if I actually read any of it [BP^2^ brochures] just because, I know it sounds silly, but I don't know if I identify as being really unhealthy.”

M16, Group 1, primipara

#### Theme 2: sources of motivation to change behaviour: “Who am I changing for?”

The mothers reported various motivating factors. Several seemed specifically motivated by increased awareness of the long-term health consequences of HDP and a desire to improve their ongoing health. In particular, they wished to minimise the risk of HDP in future pregnancies. A few were already well-informed about improving their health behaviours, although other interviewees attributed this knowledge to BP^2^.

“So, once I was told that I'm seven times more likely to have it again, I'm like ‘let's lower that percentage’... So, I did want to lose weight because I knew that I had gained weight during pregnancy. And also, I just wanted to lower the risk of any heart disease or the preeclampsia re-occurring.”

M31, Group 3, primipara

Compared with mothers who were motivated by their own health considerations, a larger proportion of the interviewees described feeling inspired by their new babies, apparent across all three groups. Many voiced a determination to adopt healthier practices now they were responsible for a new life.

“It definitely was helpful having him [baby] as an incentive knowing that what I'm putting in myself is going to him. I think that was a big driver for me to feel like I'm not just doing this for me.”

M27, Group 1, primipara

“I think looking after me at the moment is looking after them [infant twins], but also if we decide to have a future pregnancy ... You just want to reduce your risk of complications for future pregnancies as well.”

M12, Group 2, primipara

“Because you think, ‘Everything's all about [baby]’, but you've got to look after yourself as well. She [BP^2^ doctor] was, like, ‘If you're not healthy, then that's going to take its toll later in life. You're not going to be as healthy to be with [baby]’."

M02, Group 3, primipara

One mother summed up the powerful inspiration that her child provided:

“I think the biggest thing for me is I made a deal with my daughter months ago that we would go outside every day ... with COVID … I was just locking myself up in the house. I was playing with her one day, she was probably about four months old, and I was just like, ‘No, you need to see the outside world. We need to go for a walk. We’re going to do this every day.’”

M25, Group 3, primipara

Some mothers were also motivated to adopt healthy behaviours for other family members. For instance, a few mentioned improving their diets to assist a partner’s health condition or weight status, role modelling healthy cooking to older children, and joint family exercise. One woman highlighted using her new knowledge to educate her own mother about healthier eating.

“I come from [ethnic group] background. So, I guess, just now preparing your own foods, or just making Mum aware – because she lives with us – just to minimize the salt that she puts in our food as well.”

M31, Group 3, primipara

However, several mothers indicated that their focus on their children’s nutrition and well-being came at the expense of their own nutrition or physical activity. Some reported that they had little time or energy to focus on their own health. A few interviewees said that their own needs “came last” in the family.

“I tend to focus a lot on making sure my [infant] daughter's fed. So, I would say I'm probably skipping meals and then just forgetting about it. And then trying to, I guess, making up for it in the evenings, I guess ... She eats better than I do, I would say. That's probably a case with a lot of mothers, I think.”

M17, Group 3, primipara

“The baby's probably number one, work's probably two, and then the exercise will come after that. I am doing it, but it's not as much as I had previously done.”

M15, Group 1, primipara

Regardless of their understanding of the importance of healthy lifestyle, some mothers stated that they were unable to sustain good nutrition and exercise regimes. They put improving their health ‘on hold’, due to the demands of new parenthood, and also challenges from childcare, work, family commitments, financial pressures or other health concerns. Some recounted that, while recognising the importance of improved health, they lacked motivation.

“In my head it's like, I want to be healthy, I want to exercise and stuff like that. But then it's the reality. I don't have motivation, and I don't have time.”

M22, Group 2, primipara

#### Theme 3: sustaining behaviour change with a new baby: “I do what is comfortable for me and with the time I've got”

Although some interviewees reported little change in their health behaviour, many recounted various improvements to their diet following HDP, such as increasing vegetable consumption, reducing intake of salt and sugar sweetened beverages, cutting out snacks and controlling portions. Many also described trying to keep physically active, especially through walking as a response to their current circumstances. Not only was walking flexible and suitable with a young baby, during the COVID-19 restrictions it was one permissible source of exercise and socialisation, with additional mental health benefits.

“That has helped because we have now developed this daily family walk that we get to have.”

M05, Group 1, multipara

Some interviewees discussed more consciously adapting their exercise regimes to fit their circumstances.

“I just do what is comfortable for me and with the time I've got. I've actually undertaken doing a squat challenge with my sister to raise money for breast cancer this month. So, we've been doing 55 squats a day since the first of March. And for someone who hasn't ever done a squat in her life, it's a big achievement…”

M33, Group 3, multipara

Some mothers also discussed trying to adapt healthy eating to having a young baby around.

“I eat rather than just grabbing what's available. And I try to do a little bit more meal prep where I can now, which I haven't really done before, just so that I'm not just eating a party pie [small pre-cooked meat pie] because it takes five minutes.”

M06, Group 1, primipara

Most participants in Group 3 spoke positively about the Get Healthy Service (GHS). They valued the convenience and flexibility of a phone-based health coaching service and the coaches’ willingness to fit in with parenting commitments. They found the advice informative, relevant and practical; some highlighted the importance of personalised information and support, tailored to their needs and circumstances. They also reported the benefits of goal setting and the accountability of regular calls.

“So, since I've started talking to the dietitian on the phone, we're, yeah, just changing things. Cutting out the Coke, so I'm not having it like every day. Chocolates ... Yeah, it just helped me with, like how to read labels and stuff, like how much it's got sugars in it and all that stuff, and Star Ratings [government health ratings on packaging] and what to use as the alternate … I don't mind the phone calls with them to ask stupid questions, like I don't get as embarrassed because it's not face-to-face. But when I don't understand something, I'm like, ‘I didn't get it’, so she explains it quite well.”

M19, Group 3, multipara

“It's really strange. This ... faceless person, but you actually do feel like ... it does make you want to achieve what you've said you're going to do, because you've openly said it to someone.”

M02, Group 3, primipara

“She helps come up with some suggestions … I'm like, ‘I don't have time to make myself a big meal. What can I do?’ She offered me some other suggestions that I can have in the cupboard, so that I will hopefully go to them over the stupid Tim Tams [chocolate biscuits].”

M26, Group 3, primipara

“[I like] the convenience, the encouragement, the non-judgment. You know, they can be very judgmental, but the lovely girl that I speak to, she's absolutely fabulous and she's always updating me with emails and she might find something, so she sends it to me, which is very helpful.”

M33, Group 3, multipara

Mothers in both intervention groups (2 and 3) were largely positive about the BP^2^ program more generally and reported that it ‘definitely made a difference’. The consultations with dietitians and physicians provided awareness about HDP, and education and motivation to help foster healthier eating and physical activity. Participants valued the access to expert clinicians, individualised support and even a sense that someone was caring for them.

“Someone's keeping me accountable, even if it's what, every six months, I think it's always in the back of my mind, that I'm going to have to have bloods done in six months’ time.”

M12, Group 3, primipara

“I mean, I'm not healthy, I don't follow any diet regime or anything. But I think since when I went to [Hospital] to the first part of this study, I met with the dietician and she said I needed to eat more vegetables, so I’m trying to do that.”

M07, Group 2, primipara

A few participants voiced concerns about aspects of the intervention. One found the pace of the coaching service too slow and repetitive; she wanted more information in each session (#04 Group 3). Another disliked phone contact and reported that she felt that, unlike a personal trainer, the coach was insufficiently directive in setting goals (“It makes it easier for me to avoid” #30 group 3). This woman stated that the program was not motivating: “it’s just made me feel bad and not want to talk to my coach”, although she found the appointment with the cardiologist “really good”. Two women indicated that they did not require the intervention to achieve their goals as they were already self-motivated to keep healthy. “I guess I would have done something very similar without the coaching, but it was nice getting that support” (#34 Group 3).

Although all BP^2^ participants received brochures about GHS, and women in Groups 1 and 2 could sign up to the six-month program, only women in Group 3 were actively enrolled by the research team into GHS. One Group 1 mother sought GHS contact, as she had already been enrolled during her preceding pregnancy; another had used GHS in pregnancy but not postnatally. Otherwise, no other Group 1 or 2 women enrolled in the service; women appeared to accept whichever follow-up strategy they were given. For instance, when asked if the BP^2^ program had influenced her motivation to be healthy, one mother stated:

“Not a huge amount of difference, but I think that possibly is because of the group I was put in, to be honest.”

M14, Group 1, primipara

Although many women were motivated to make changes to improve their lifestyle and long-term health, it appeared that without the structured follow-up built in to all BP^2^ groups, women were unlikely to seek out support independently:

“You guys, the research team, they connected me with the Get Healthy. And if it wasn't for that I don't think that I'd be where I'm at right now”

M31, Group 3, primipara

## Discussion

This study demonstrates that 10–12 months after a hypertensive pregnancy, some women reported difficulties in altering their health behaviour, but many indicated that they had adopted healthier eating or exercise habits – to varying degrees. Most of those in the BP^2^ groups receiving ongoing education and lifestyle coaching (2 and 3) reported positive changes. However, there was minimal evidence of women seeking additional support (e.g. attending GHS independently) beyond the structure of whichever of the BP^2^ groups they were assigned to. We therefore argue that structured and routine post-HDP interventions such as provided to the BP^2^ intervention groups 2 and 3 are needed to provide motivation and personalised support to facilitate behaviour change.

Early parenthood is clearly a time of substantial change for most women. These participants had experienced HDP, resulting in significant consequences for their future health, in addition to the pressures of caring for a young baby. While likely to benefit from more general interventions to promote healthy behaviour postnatally, the three themes identified in the qualitative analysis demonstrate important elements in supporting this group specifically.

Despite its significance as a CVD risk factor, there is limited awareness among women of the role that HDP plays [13, 17, 20, 31]. This was apparent among BP^2^ participants; several stated that they had not understood the implications of HDP until they enrolled. A few in the usual care group (Group 1) assimilated information from BP^2^ brochures which prompted them to focus on their health status. Yet, others in this group did not seem as aware of the risks or the association with diet and exercise. In contrast, several in Groups 2 and 3 who visited the clinicians at six months clearly absorbed the significance of the condition for their future health, and two described the revelation as a “wake-up call” (M01 and M10). This finding has implications not only for future interventions, but for general antenatal, birthing and primary health services that have not, to date, conveyed the seriousness of HDP for women’s ongoing health [18]. This may be particularly important for women who experienced fewer HDP complications, with prior research demonstrating better risk perception among women with severe disease or whose infants were born preterm [31], and among those with a family history of CVD [32].

Although important, awareness of future health risks alone is rarely sufficient motivation to change behaviour. One study of women after preeclampsia found that communicating about risk factors changed their intention to stop smoking, but not to increase their exercise or eat more healthily [33]. Previous qualitative studies with women who had experienced HDP identified not only limited recognition of the association with future CVD, but also a desire for specific support to adopt healthier lifestyles [19, 20]. This highlights the difficulties of sustaining behaviour change without support. One qualitative study explored motivators and barriers to healthy postpartum lifestyle changes among 36 Dutch women who had experienced preeclampsia, gestational diabetes and/or intrauterine growth restriction during pregnancy. Despite positive intentions, many women were unable to maintain a healthy lifestyle, often related to limited knowledge and professional support, and poor physical and emotional recovery after the birth [34]. This suggests that other factors also influence personal motivation in this population.

In this study, interviewees revealed varying levels and sources of motivation to adopt healthier behaviour. In terms of the socio-environmental model, some women seemed sufficiently motivated at the individual level. They had a personal determination to maintain or adopt a healthy lifestyle, driven by an individual commitment to good health and the knowledge that they faced the additional hurdle of a history of HDP. A larger group appeared strongly motivated by interpersonal relationships, particularly their role in raising their new infants. These women were focused on providing optimum nutrition and environments for their babies, and on staying healthy to nurture and enjoy them. Reducing the risk of HDP in future pregnancies and the risk to other infants was also a common incentive for behaviour change. For some women, however, knowledge gained through BP^2^ appeared to be insufficient to motivate sustained behaviour change in the context of early parenthood. For this group and those who were too overwhelmed by parenting and other responsibilities to prioritise their own health (albeit a minority in this sample), interventions in the first postpartum year like BP^2^ are unlikely to have impact. Support should focus on maintaining primary care ongoing follow-up and assessment of readiness for change [35]. At a societal level, there was evidence of the impact of organisations such as the GHS and the wider BP^2^ program in motivating behaviour change, by raising awareness and providing access to tailored information, encouragement and follow-through.

Further, participation in the structured intervention offered many mothers the support, knowledge and resources to adopt and maintain healthy behaviour. Some women specifically cited the accountability to others through regular monitoring and follow-up contact as a motivating factor. Discussions with health professionals, including sustained contact with health coaches, helped them learn new ways to be healthy. Interviewees particularly valued personalised health advice, such as combining food preparation for baby and family, dealing with cravings or finding suitable fitness regimes. Many described adapting healthy behaviour to the constraints of having a young baby or the COVID pandemic, demonstrating the role of health professional advice during the critical early months of parenting.

### Implications for practice

Our interview findings suggest the importance of structured follow-up, tailored to individual women’s needs and circumstances, in enhancing knowledge and facilitating behaviour change following HDP. The more intense intervention (face-to-face consultation with a specialist medical practitioner and dietitian followed by a six-month telephone-based coaching program—Group 3) appeared to be more successful in motivating women to adopt healthy behaviours and to be accountable for their health behaviour than information brochures and GP follow-up. This finding is not surprising, as surveyed Australian GPs have only moderate knowledge regarding post-HDP health and expressed a need for further education themselves [14]. However, GPs are usually the practitioner ultimately looking after the ongoing health of women post-HDP and their children, although contact may be less frequent than during pregnancy. Therefore, their incorporation into post-HDP follow-up systems is vital. Potentially, a graduated transfer of care back to primary healthcare during the first postpartum year may be appropriate, with a specialised postpartum clinic and GHS offered to all women 6–12 months postpartum to facilitate early intervention while they are receptive to this, with detailed correspondence to the woman’s GP and ongoing GP follow-up thereafter. Interventions and consumer education should seek to motivate behaviour not only by focusing not only on women’s desire for improved future health, but also their commitment to future pregnancies and their children’s ongoing needs for a healthy family environment, potentially engaging other primary healthcare services such as child and family health nurses. Such interventions could improve health outcomes for other groups of postnatal women and their children, such as those who had overweight or obesity antenatally or who experienced excessive gestational weight gain. It is also important to recognise that, as found in this study, no “one size fits all”, and women not motivated to engage in the year postpartum still require periodic follow-up for CVD risk factor assessment and education when willing to do so.

### Strengths and limitations

A major strength of this study is its incorporation within a sizeable, randomised trial, BP^2^, which to the best of our knowledge, is the largest RCT to date focussed on structured early follow-up and lifestyle behaviour change amongst women following HDP. The 34 mothers interviewed in this qualitative sub-study included women across all intervention groups and the broader BP^2^ study’s sociodemographic range. Although this group was broadly typical of the overall BP^2^ cohort, interviewees were more likely to be first-time parents. Potentially, these mothers may be more likely to be able to engage in lifestyle behaviour change than women with more than one child.

It is possible that women who volunteered were more positive about the program or had engaged more actively in the proposed lifestyle changes. Although the interviewer was not involved in other aspects of the BP^2^ intervention, at least one respondent appeared to think that she was a member of the overall study team. This perception may have influenced participants’ responses and biased them to respond more favourably about the program or to overstate their healthy behaviours. However, two interviewees voiced some reservations about aspects of the GHS and a number described making few changes in their health behaviour. Additionally, women participating in the broader BP^2^ RCT may not themselves be representative of the general post-HDP population, particularly regarding higher education status and health literacy. However, since we found considerable post-HDP health knowledge gaps and high need for structured education and support even in this majority university educated cohort, this may not be a major limitation.

## Conclusions

Among participants in an early post-HDP RCT aimed at improving women’s health, the majority demonstrated recognition of their future health risks and motivation to improve their lifestyle. This was particularly true for women in the highest intensity intervention group, who emphasised the importance of the intervention in motivating and supporting their lifestyle behaviour change given their increased risk of CVD. Others were motivated by their commitment to optimising their infants’ future well-being, a trend common to more general postnatal populations. These findings suggest that merely offering options that women are unlikely to proactively embrace during the challenges of new parenthood will be insufficient to promote sustained behaviour change. Rather, this study highlights the value of targeted, structured, routine support to assist women to adopt and/or maintain healthy lifestyles following HDP.

## Supplementary Information

**Additional file 1. **Interview questions x BP^2^ study arm.

## Data Availability

The transcripts analysed during this qualitative sub-study are available from the corresponding author on reasonable request. However, the overall BP^2^ dataset is not available as the study is still ongoing.

## Abbreviations

AUD: Australian dollars
BP^2^: Blood Pressure Postpartum Study
CH: Chronic hypertension
CVD: Cardiovascular disease
GH: Gestational hypertension
GHS: Get Healthy Service
GP: General practitioner
HDP: Hypertensive disorders of pregnancy
PE: Preeclampsia
RCT: Randomised controlled trial
