# Supplementary material for: Optimising mothers’ health behaviour after hypertensive disorders of pregnancy: a qualitative study of a postnatal intervention
Source: BMC Public Health. 2022 Jun 27;22:1259. doi: 10.1186/s12889-022-13590-2 (PMC9235190; doi:10.1186/s12889-022-13590-2)
Supplement: Supplementary file 1 — Additional file 1. Interview questions x BP2 study arm. [file 12889_2022_13590_MOESM1_ESM.docx]

**INTERVIEW QUESTIONS x ARM**

**ARM 1 – Extended lifestyle intervention. Info pack (Get Healthy Service, Heart Foundation and My Baby Now app)**

Thank you very much for agreeing to this interview. As you may know we are interviewing women who are taking part in the Blood Pressure Postpartum study. We are talking to women who have had high blood pressure in pregnancy to find out what things make it harder and easier to keep healthy. We are also interested in your feedback about being in the Blood Pressure Postpartum study.

I would just like to confirm that you are happy for me to record this interview?

I realise that you have filled in various questionnaires, but I don’t have access to that information. I know that you had a little boy/girl last year, but I don’t know their name. I’d like to use a name in the interview, so you can tell me their name if you like or use a pseudonym. I realise you may have to leave the interview to attend to him/her. That’s fine – I can always call you back later. And please remember you can skip over any questions you don’t feel comfortable to answer.

| **First of all, I’d like to ask you about your diet and eating habits….** | **SEM Domain** |
| --- | --- |
| 1. Have your eating habits changed since you had your baby? 2. How has your diet changed since having (baby name)? 3. How does that compare with when you were pregnant? 4. How does that compare with before you were expecting (baby name)? 5. [If mention changes in diet] Have these changes had an impact on your everyday life? How? 6. [If mention changes in diet] How have these changes affected your family or the people you live with? 7. Have you faced any challenges in eating a healthy diet? [You already mentioned xxxx, is there anything else that makes it hard?] 8. Has anything helped you in eating a healthy diet? Have you received support from your family or friends? 9. Do you feel that eating healthy food is important to your friends and family? | Individual  Interpersonal  Interpersonal |
| **Now I have some questions about physical activity ….** |  |
| 1. Have your exercise habits changed since you had your baby? 2. How has your level of physical activity changed since having (baby name)? 3. How does that compare with when you were pregnant? 4. How does that compare with before you were expecting (baby name)? 5. [If mention changes in PA] Have these changes had an impact on your everyday life? How? 6. [If mention changes in PA] How have these changes affected your family or the people you live with? 7. Have you faced any challenges in being physically active? [You already mentioned xxxx, are there other factors that make it hard?] 8. Has anything helped you in keeping active? Have you received support from your family or friends? 9. Do you feel that being physically active is important to your friends and family? | Individual  Interpersonal  Interpersonal |
| **Looking now at healthy lifestyle in general….**   1. Have you experienced any challenges to other aspects of your health since you had (baby name)? Like smoking or drinking? 2. If so, did this involve your family or friends? | Individual  Interpersonal |
| 1. How motivated are you to eat healthy food and participate in physical activity? 2. Has participating in the BP^2^ program made a difference to your motivation? | Individual  Organisation |
| 1. Do you feel confident that you can maintain the lifestyle changes you’ve made (if any and if positive)? 2. Can you think of anything that might make it easier (or harder) for you to maintain these changes? 3. Have you found any groups or services in your local area that have helped you adopt a healthy lifestyle and maintain a healthy weight? 4. What do you think organisations could do to help mums like you to eat well and be physically active? Like workplaces or local councils? 5. Could governments do anything? | Individual  Community  Policy |
| **Now I’m going to ask about your contact with health professionals** |  |
| 1.1 Have you discussed cardio-vascular health with your GP or practice nurse? Have you discussed diet and PA? Can you tell me a bit about the information they gave you? Was there anything that was particularly helpful? | Community |
| 1.2 Do you recall receiving an information pack from the BP^2^ team about keeping healthy after having high blood pressure in pregnancy? You would have received that after you signed up for the blood pressure research, when (baby name) was about six months.  **If YES**, was it helpful? | Organisation |
| 1.3 Do you remember receiving a flyer about the NSW Get Healthy Service?  1.4 Did you contact the GHS?  If NO – stop interview. No further questions.  If YES 1.5 What made you decide to join GHS? (PROMPT: goals?)  1.6. How many calls do you recall with your health coach? Was it the same coach each time?  1.7 Did the GHS calls suit you? (PROMPT: number of calls, timing of calls, convenience, fitting with parenting routines, cultural appropriateness). Did it suit your family?  1.8 What did you like about the GHS? What did you dislike? (PROMPT: Talking on the phone about your health, feelings about health coach – gender?)  1.9 Has being part of the GHS helped you? How?  1.10 If there was anything about GHS that you would change, what would that be? | Organisation |

**That is the end of my questions… unless there is anything else you would like to add about what we’ve been talking about.**

**Thank you so much for giving me the time for this interview. We will send your gift voucher in the next week or so.**

**ARM 2 – Extended lifestyle intervention. Info pack (Get Healthy Service, Heart Foundation and My Baby Now app) + visit to follow up clinic at 6mth – discussion with doctor and dietitian**

Thank you very much for agreeing to this interview. As you may know we are interviewing women who are taking part in the Blood Pressure Postpartum study. We are talking to women who have had high blood pressure in pregnancy to find out what things make it harder and easier to keep healthy. We are also interested in your feedback about being in the Blood Pressure Postpartum study.

I would just like to confirm that you are happy for me to record this interview?

I realise that you have filled in various questionnaires, but I don’t have access to that information. I know that you had a little boy/girl last year, but I don’t know their name. I’d like to use a name in the interview, so you can tell me their name if you like or use a pseudonym. I realise you may have to leave the interview to attend to him/her. That’s fine – I can always call you back later. And please remember you can skip over any questions you don’t feel comfortable to answer.

| **First of all, I’d like to ask you about your diet and eating habits….** | **SEM Domain** |
| --- | --- |
| 1. Have your eating habits changed since you had your baby? 2. How has your diet changed since having (baby name)? 3. How does that compare with when you were pregnant? 4. How does that compare with before you were expecting (baby name)? 5. [If mention changes in diet] Have these changes had an impact on your everyday life? How? 6. [If mention changes in diet] How have these changes affected your family or the people you live with? 7. Have you faced any challenges in eating a healthy diet? [You already mentioned xxxx, is there anything else that makes it hard?] 8. Has anything helped you in eating a healthy diet? Have you received support from your family or friends? 9. Do you feel that eating healthy food is important to your friends and family? | Individual  Interpersonal  Interpersonal |
| **Now I have some questions about physical activity ….** |  |
| 1. Have your exercise habits changed since you had your baby? 2. How has your level of physical activity changed since having (baby name)? 3. How does that compare with when you were pregnant? 4. How does that compare with before you were expecting (baby name)? 5. [If mention changes in PA] Have these changes had an impact on your everyday life? How? 6. [If mention changes in PA] How have these changes affected your family or the people you live with? 7. Have you faced any challenges in being physically active? [You already mentioned xxxx, are there other factors that make it hard?] 8. Has anything helped you in keeping active? Have you received support from your family or friends? 9. Do you feel that being physically active is important to your friends and family? | Individual  Interpersonal  Interpersonal |
| **Looking now at healthy lifestyle in general….**   1. Have you experienced any challenges to other aspects of your health since you had (baby name)? Like smoking or drinking? 2. If so, did this involve your family or friends? | Individual  Interpersonal |
| 1. How motivated are you to eat healthy food and participate in physical activity? 2. Has participating in the BP^2^ program made a difference to your motivation? | Individual  Organisation |
| 1. Do you feel confident that you can maintain the lifestyle changes you’ve made (if any and if positive)? 2. Can you think of anything that might make it easier (or harder) for you to maintain these changes? 3. Have you found any groups or services in your local area that have helped you adopt a healthy lifestyle and maintain a healthy weight? 4. What do you think organisations could do to help mums like you to eat well and be physically active? Like workplaces or local councils? 5. Could governments do anything? | Individual  Community  Organisation  Policy |
| **Now I’m going to ask about your contact with health professionals** |  |
| 2.1 Have you discussed cardio-vascular health with your GP or practice nurse? Have you discussed diet and PA? Can you tell me a bit about the information you received? Was there anything that was particularly helpful? | Community |
| 2.2 Do you recall receiving an information pack from the BP^2^ team about keeping healthy after having high blood pressure in pregnancy? You would have received that after you signed up for the blood pressure research, when (baby name) was about six months.  **If YES**, 2.3 Was it helpful? | Organisation |
| 2.4 Do you remember receiving a flyer about the NSW Get Healthy Service?  If **NO** – go to Q2.12  2.5 Did you contact the GHS? If **NO** – Go to Q2.12  If **YES**  2.6 what made you decide to join GHS? (PROMPT: goals?)  2.7 How many calls do you recall with your health coach? Was it the same coach each time?  2.8 Did the GHS calls suit you? (PROMPT: number of calls, timing of calls, convenience, fitting with parenting routines, cultural appropriateness). Did it suit your family?  2.9 What did you like about the GHS? What did you dislike? (PROMPT: Talking on the phone about your health, feelings about health coach – gender?)  2.10 Has being part of the GHS helped you? How?  2.11 If there was anything about GHS that you would change, what would that be? | Organisation |
| 2.12 Did you visit the hospital clinic as part of the BP2 study, when your baby was about 6 months old?  If **YES** Can you tell me a bit about the visit? Was there anything you particularly remember about the visit? Or any helpful information or advice you received? | Organisation |

**That is the end of my questions… unless there is anything else you would like to add about what we’ve been talking about. Thank you so much for giving me the time for this interview. We will send your gift voucher in the next week or so.**

**ARM 3 – Extended lifestyle intervention. Info pack (Get Healthy Service, Heart Foundation and My Baby Now app) + visit to follow up clinic at 6mth – discussion with doctor and dietitian + lifestyle behaviour coaching through Get Healthy Service.**

Thank you very much for agreeing to this interview. As you may know we are interviewing women who are taking part in the Blood Pressure Postpartum study. We are talking to women who have had high blood pressure in pregnancy to find out what things make it harder and easier to keep healthy. We are also interested in your feedback about being in the Blood Pressure Postpartum study.

I would just like to confirm that you are happy for me to record this interview?

I realise that you have filled in various questionnaires, but I don’t have access to that information. I know that you had a little boy/girl last year, but I don’t know their name. I’d like to use a name in the interview, so you can tell me their name if you like or use a pseudonym. I realise you may have to leave the interview to attend to him/her. That’s fine – I can always call you back later. And please remember you can skip over any questions you don’t feel comfortable to answer.

| **First of all, I’d like to ask you about your diet and eating habits….** | **SEM Domain** |
| --- | --- |
| 1. Have your eating habits changed since you had your baby? 2. How has your diet changed since having (baby name)? 3. How does that compare with when you were pregnant? 4. How does that compare with before you were expecting (baby name)? 5. [If mention changes in diet] Have these changes had an impact on your everyday life? How? 6. [If mention changes in diet] How have these changes affected your family or the people you live with? 7. Have you faced any challenges in eating a healthy diet? [You already mentioned xxxx, is there anything else that makes it hard?] 8. Has anything helped you in eating a healthy diet? Have you received support from your family or friends? 9. Do you feel that eating healthy food is important to your friends and family? | Individual  Interpersonal  Interpersonal |
| **Now I have some questions about physical activity ….** |  |
| 1. Have your exercise habits changed since you had your baby? 2. How has your level of physical activity changed since having (baby name)? 3. How does that compare with when you were pregnant? 4. How does that compare with before you were expecting (baby name)? 5. [If mention changes in PA] Have these changes had an impact on your everyday life? How? 6. [If mention changes in PA] How have these changes affected your family or the people you live with? 7. Have you faced any challenges in being physically active? [You already mentioned xxxx, are there other factors that make it hard?] 8. Has anything helped you in keeping active? Have you received support from your family or friends? 9. Do you feel that being physically active is important to your friends and family? | Individual  Interpersonal  Interpersonal |
| **Looking now at healthy lifestyle in general….**   1. Have you experienced any challenges to other aspects of your health since you had (baby name)? Like smoking or drinking? 2. If so, did this involve your family or friends? | Individual  Interpersonal |
| 1. How motivated are you to eat healthy food and participate in physical activity? 2. Has participating in the BP^2^ program made a difference to your motivation? | Individual  Organisation |
| 1. Do you feel confident that you can maintain the lifestyle changes you’ve made (if any and if positive)? 2. Can you think of anything that might make it easier (or harder) for you to maintain these changes? 3. Have you found any groups or services in your local area that have helped you adopt a healthy lifestyle and maintain a healthy weight? 4. What do you think organisations could do to help mums like you to eat well and be physically active? Like workplaces or local councils? 5. Could governments do anything? | Individual  Community  Policy |
| **Now I’m going to ask about your contact with health professionals** |  |
| 3.1 Have you discussed cardio-vascular health with your GP or practice nurse? Have you discussed diet and PA? Can you tell me a bit about the information you received? Was there anything that was particularly helpful? | Community |
| 3.2 Do you recall receiving an information pack from the BP^2^ team about keeping healthy after having high blood pressure in pregnancy? You would have received that after you signed up for the blood pressure research, when (baby name) was about six months.  **If YES**, was it helpful? | Organisation |
| 3.3 Did you visit the hospital clinic as part of the BP^2^ study, when your baby was about 6 months old?  Can you tell me a bit about the visit? Was there anything you particularly remember about the visit? Or any helpful information or advice you received? | Organisation |
|  |  |
| 3.5 Have you been in touch with the Get Healthy Service?  **If NO**, why is that? AND STOP Interview. No further questions  **If YES**.  3.6 When you were enrolled in the GHS, what did you hope to get out of the program? (PROMPT: goals?)  3.7 How many calls do you recall with your health coach? Was it the same coach each time?  3.8 Did the GHS calls suit you? (PROMPT: number of calls, timing of calls, convenience, fitting with parenting routines, cultural appropriateness). Did it suit your family?  3.9 What did you like about the GHS? What did you dislike? (PROMPT: Talking on the phone about your health, feelings about health coach – gender?)  3.10 Has being part of the GHS helped you? How?  3.11 If there was anything about GHS that you would change, what would that be? | Organisation |

**That is the end of my questions… unless there is anything else you wish to add about what we’ve been talking about. Thank you so much for giving me the time for this interview. We will send your gift voucher in the next week or so.**
